# Identifying diabetes from conjunctival images using a novel hierarchical multi-task network

**DOI:** 10.1038/s41598-021-04006-z

**Published:** 2022-01-07

**Authors:** Xinyue Li, Chenjie Xia, Xin Li, Shuangqing Wei, Sujun Zhou, Xuhui Yu, Jiayue Gao, Yanpeng Cao, Hong Zhang

**Affiliations:** 1 Eye Hospital, The First Affiliated Hospital of Harbin Medical University, No.143, Yiman Street, Nangang District, Harbin City, 150001 Heilongjiang Province China; 2 Key Laboratory of Basic and Clinical Research of Heilongjiang Province, Harbin, 150001 China; 3 Eye Department, Shanghai Children ‘s Hospital, Shanghai Jiaotong University, Shanghai, China; 4 State Key Laboratory of Fluid Power and Mechatronic Systems, School of Mechanical Engineering, Zhejiang University, Room 230, Building 1, Yuquan Campus, 38 Zhe Da Road, Hangzhou, 310027 Zhejiang Province China; 5 School of Electrical Engineering and Computer Science, 2002 Digital Media Center, Louisiana State University, 340 E. Parker Blvd, Baton Rouge, LA 70803 USA

**Keywords:** Type 2 diabetes, Eye manifestations, Electrical and electronic engineering, Eye abnormalities, Computer science

## Abstract

Diabetes can cause microvessel impairment. However, these conjunctival pathological changes are not easily recognized, limiting their potential as independent diagnostic indicators. Therefore, we designed a deep learning model to explore the relationship between conjunctival features and diabetes, and to advance automated identification of diabetes through conjunctival images. Images were collected from patients with type 2 diabetes and healthy volunteers. A hierarchical multi-tasking network model (HMT-Net) was developed using conjunctival images, and the model was systematically evaluated and compared with other algorithms. The sensitivity, specificity, and accuracy of the HMT-Net model to identify diabetes were 78.70%, 69.08%, and 75.15%, respectively. The performance of the HMT-Net model was significantly better than that of ophthalmologists. The model allowed sensitive and rapid discrimination by assessment of conjunctival images and can be potentially useful for identifying diabetes.

## Introduction

Deep learning has been successfully applied in the field of medical image analysis. Because of its high learning ability and computing ability, deep learning can even extract hidden information that doctors cannot perceive from the data. For ophthalmic image analysis, deep learning can detect several systemic diseases through eye images, such as anemia^1^, cardiovascular disease^2^, hepatobiliary disease^3^, traumatic brain injury^4^ etc. This is because the visual blood vessels and nerves in the eyes are closely related to the health of the whole body. Diabetes is a chronic disease involving multiple systems. It is one of the fastest growing public health problems in the twenty-first century, affecting 463 million people around the world^5^. Several studies have shown that hyperglycemia first damages smaller blood vessels such as the bulbar conjunctival microcirculatory system. The bulbar conjunctiva is the vascularized mucosa that covers the surface of the eyeball. Since bulbar conjunctiva is the only blood vessel in the body that can be seen directly without using any equipment, bulbar conjunctival vessels can be used as a feasible window for studying diabetes. Since 1956, researchers have developed several imaging techniques such as computer-assisted intravital microscopy (CAIM)^6,7^, EyeFlow^8,9^, and Retinal Function Imager to observe conjunctival hemodynamic changes in patients with diabetes^10^. These studies found that conjunctival vessels showed similar changes as in diabetic retinopathy, such as dilation and distortion. However, the analytical methods used in these studies are time-consuming and insufficiently objective, which limits their potential to be developed into diagnostic indicators.

In this study, we intend to explore the possibility of extracting disease signals from conjunctival images of patients with diabetes through deep learning. However, key challenges in developing an effective deep learning system are that a large number of annotated training samples are needed for supervised learning^11,12^, and the high cost to obtain enough high-quality, clinical samples. To overcome these challenges, we developed a novel and personalized algorithm called the hierarchical multi-task network (HMT-Net) for identifying diabetes using conjunctival images. We creatively integrated ophthalmologists’ professional knowledge into the deep network design and trained an effective recognition system using limited available datasets.

Specifically, based on previous observation of diabetic conjunctiva (e.g., Ditzel^13^, Khan^14^, and Khansari^15^, Shahidi^16^, our experience, and the characteristics of conjunctival images, our models are designed in the deep network to answer four groups of sub-questions related to diabetic conjunctival abnormalities, and then the results of these sub-problems are integrated for the final judgment of diabetes. This algorithm can overcome data insufficiency and is sufficiently accurate compared with traditional models. It also reduces the dependence on big datasets and can be conveniently applied to the medical setting.

## Methods

**Figure 1:**
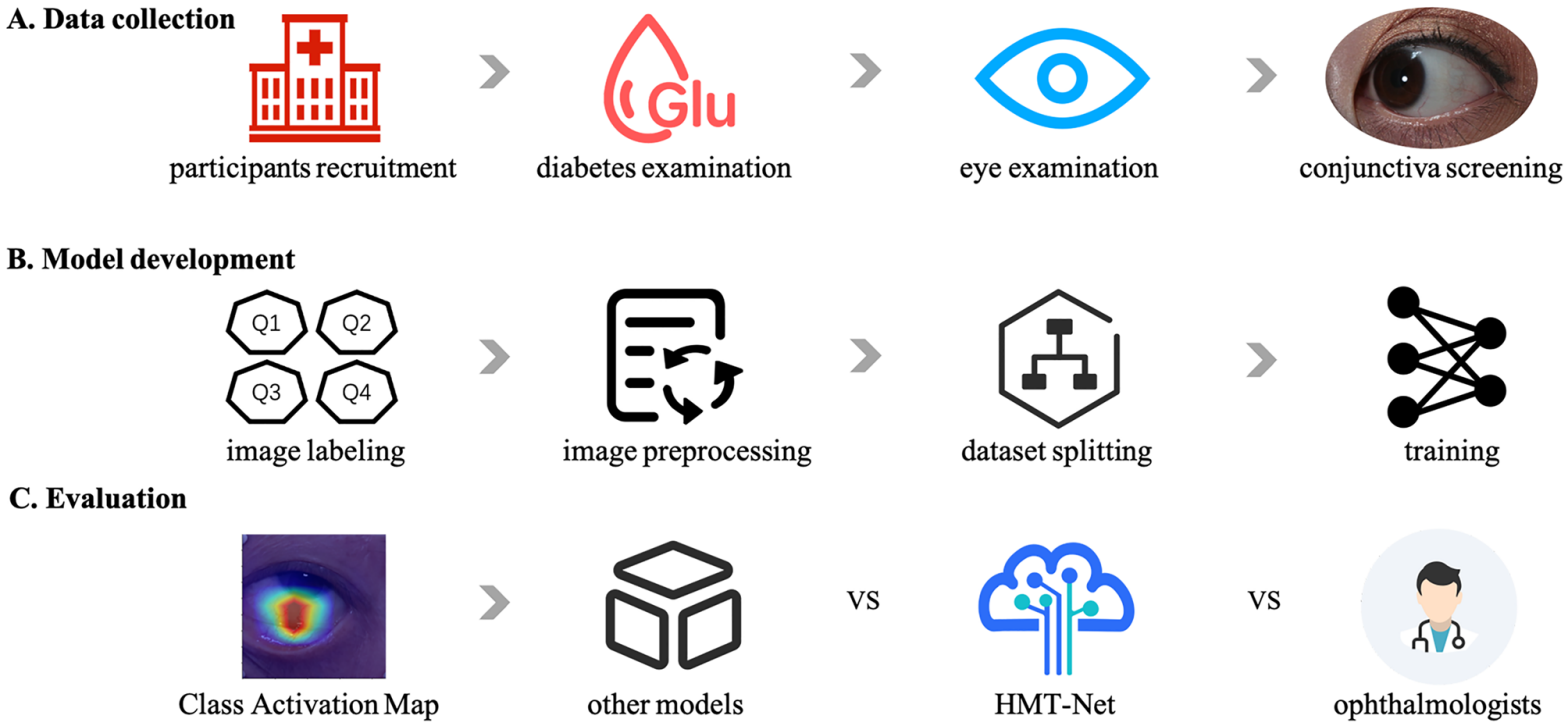
Overview of the study.

The overall flow chart of the experiment is shown in Fig. 1. The study flow is divided into three stages: data collection, model development, and model evaluation.

### Data collection

#### Study participants

This was a single-center, prospective, diagnostic, and exploratory study. The data were collected from the First Affiliated Hospital of Harbin Medical University. The disease group consisted of patients with type 2 diabetes diagnosed in the endocrinology department of the hospital. The control group was composed of non-diabetic healthy volunteers from the physical examination center of the hospital. All subjects underwent detailed and standardized eye examinations and diabetes-related examinations. Conjunctival images and clinical history were collected. Patients who met at least one of the exclusion criteria (Supplementary Document p1) were excluded.

All procedures followed the tenets of the Declaration of Helsinki. Written informed consent was obtained from each participant. The study was approved by the Ethics Committee of the First Affiliated Hospital of Harbin Medical University (2020130). This study was prospectively registered at Chinese Clinical Trials Registry (ChiCTR2000037498).

#### Image acquisition

The eyes of all the selected subjects were examined in detail. All the images were taken by the same technician using a digital camera (EOS 5D Mark II, Canon, Tokyo, Japan) in the same darkroom environment (more details in Supplementary Document p2).

### Model development

#### Image labeling

According to the previous summary of diabetic conjunctival changes, four related sub-questions (Q1 ~ Q4) were designed to guide the recognition of diabetes. The collected conjunctival images were given corresponding labels for each question of Q1 ~ Q5 (Q5: whether or not diabetic). Based on other studies of conjunctival images and characteristics of the conjunctival images, the classification criteria were further established (Supplementary Document p3-4, Figure S1). Three well-trained ophthalmologists strictly followed the classification criteria and labeled each conjunctival image. During the process, some poor quality images (e.g. images with eyelid occlusion more than 1/10 of the whole image, low contrast, or blurred focus) were excluded.

#### Image preprocessing and data augmentation

The original image data required certain image preprocessing operations and dataset augmentation before being input into the network. See Figure S2 for more details (Supplementary Document p5-8).

#### Dataset splitting

We adopt cross validation to utilize these images. We divided the data set into 5 parts (from Set1 to Set5) and then selected one part of the sample as the test set each time, and then used the remaining samples for training.

#### System Design and Training

**Figure 2:**
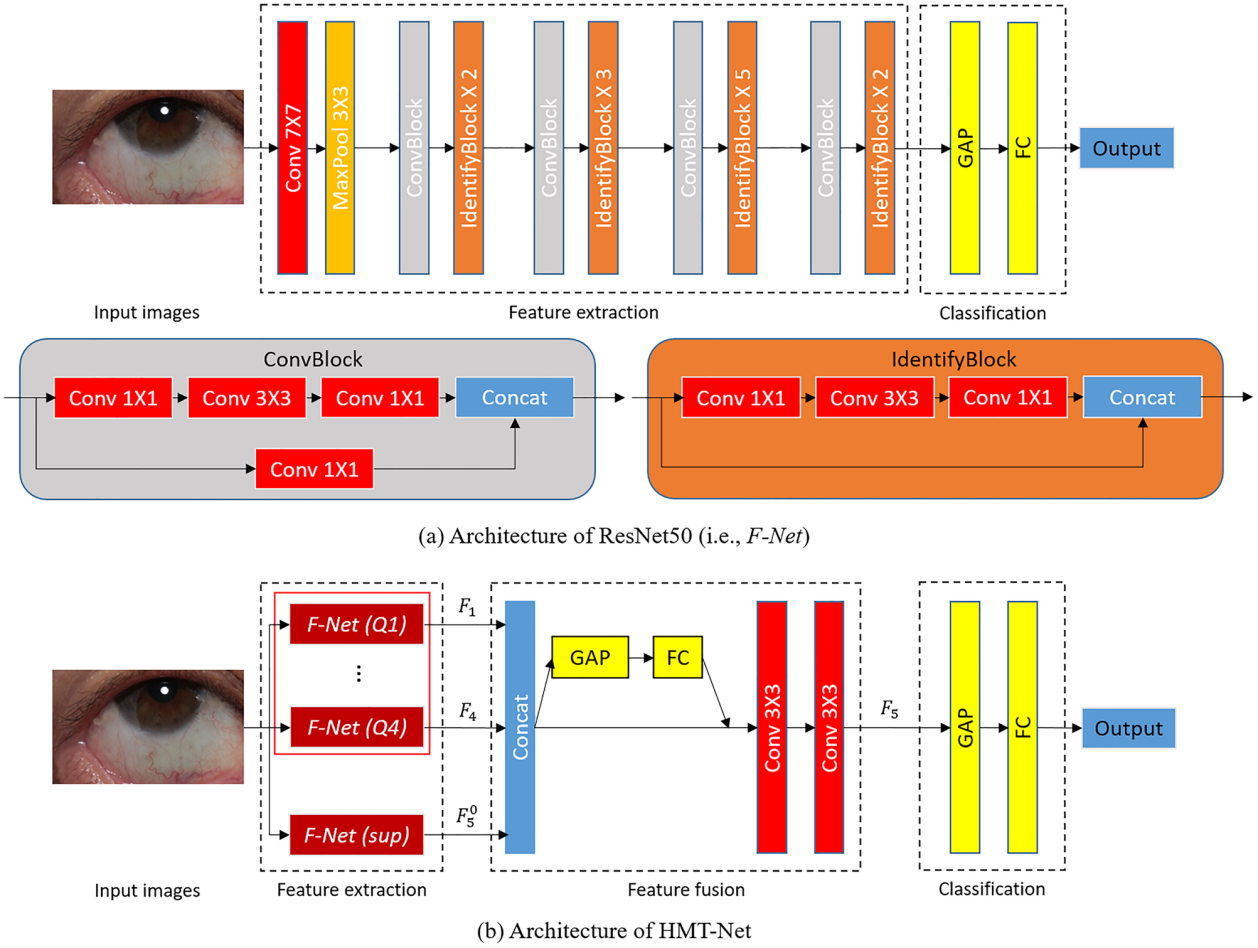
Architectures of ResNet50 and HMT-Net. (**a**) Network architecture for F-Net (Q1) ~ F-Net (Q4). (**b**) Integrating F-Nets (with GAP and FC layers removed) into HMT-Net. GAP = Global Average Pooling. FC = Fully Connected.

The HMT-Net was trained through two stages (Fig. 2).

1. In Stage-I, we chose ResNet50 as the backbone, and then performed transfer learning based on ImageNet and a portion of our labeled dataset (Dataset-I) to train four sub-networks and answer four sub-questions. Four feature extraction sub-networks $F\text{-}Net (Q1)$~ $F\text{-}Net (Q4)$ were trained using the ophthalmologist-designed classification tasks of Q1 ~ Q4.
2. In Stage-II, we hierarchically integrated four previously trained sub-networks $F{-}Net (Q1)$~ $F\text{-}Net (Q4)$ to build an HMT-Net and trained the HMT-Net using another portion of our labeled dataset (Dataset-II) for identifying diabetes (Q5).

**Q1 ~ Q4 classification tasks.** $F\text{-}Net (Q1)$~ $F\text{-}Net (Q4)$ were built using ResNet50^17^ as a backbone structure, and trained for Q1 ~ Q4. ResNet50 is a widely used deep architecture for feature extraction and classification. ResNet50, which outperforms other widely used architectures such as VGG19^18^ and MobileNet^19^ in Q1 ~ Q4 classification, was adopted through experiments. The architecture of such an $F\text{-}Net$ is shown in Fig. 2a. To overcome the issue of insufficient amount of labeled data, transfer learning technique was utilized by pre-training these networks using the big general image dataset ImageNet^20^ and then fine-tuning using Dataset-I.

**Q5 diabetes classification task.** The purpose of Stage-II was to utilize sub-task classifications $F\text{-}Net (Q1)$~ $F\text{-}Net (Q4)$ trained in Stage-I to construct the HMT-Net for final diabetes classification of Q5. The HMT-Net was trained and fine-tuned using Dataset-II. The architecture of the HMT-Net is shown in Fig. 2b, which mainly includes three parts: feature extraction network, feature fusion network, and classification network.

The feature extraction network consisted of five parallel branches. The first four were from $F\text{-}Net (Q1)$~ $F\text{-}Net (Q4)$, whose feature extraction layers were kept while Global Average Pooling (GAP) and Fully Connected (FC) layers were removed. In order to utilize the features extracted for Q1 ~ Q4 in $F\text{-}Net (Q1)$ ~ $F\text{-}Net (Q4)$, the parameters of these four branches were kept during Stage-II (training for Q5). For Q5, another branch was added following the structure of $F\text{-}Net$ (without GAP and FC) and denoted as $F\text{-}Net (sup)$. Unlike the first four branches, the parameters of $F\text{-}Net (sup)$ were pre-trained using ImageNet^20^ and then fine-tuned using Dataset-II during Stage-II. $F\text{-}Net (sup)$ generated supplementarily (to ${F}_{1}$ ~ ${F}_{4}$) features ${F}_{5}^{0}$ for Q5. Thus, the entire feature extraction network output ${F}_{1}$ ~ ${F}_{4}$ and ${F}_{5}^{0}$.

The feature fusion network integrated these complementary features to generate more discriminative features for the Q5 diabetes classification task. First, ${F}_{1}$ ~ ${F}_{4}$ and ${F}_{5}^{0}$ were concatenated to obtain the combined features. Then, adaptive learning^21^ was used to compute channel attentions, which assigned suitable weights to different channels of features. Using two consecutive convolutions (with kernel size 3 and stride 1, the numbers of convolution kernels were 4096 and 2048, respectively), the dimension of the features was compressed from 10,240 to 2048. The experimental results showed that redundant information in the concatenated feature maps could be compressed to generate more discriminative features for the subsequent Q5 classification.

Finally, a classification network mapped the processed features to the category of Q5. This network was composed of a GAP and an FC layer, which integrated relevant information with class discrimination. The final output (identify outcome) was obtained using a softmax function.

The network was implemented using PyTorch^22^ and the cross-entropy loss function was adopted. The parameters of the network were updated through backpropagation. (more details in Supplemental Document-p9, Figure S3).

### System Evaluation

#### Performance metrics

The model accuracy was evaluated by using three different metrics.

Sensitivity (Recall): $$SE=TP/(TP+FN)$$,

Specificity: $$SP=TN/(TN+FP)$$,

Accuracy: $$ACC=(TP+TN)/(TP+TN+FP+FN)$$,

where TP, FP, TN, and FN represent the numbers of true positives, false positives, true negatives, and false negatives, respectively. Diabetes and health were defined as positive and negative samples respectively.

The receiver operating characteristic (ROC) curve and the area under ROC curve (AUC) were also computed to comprehensively evaluate the classification models of Q5 under Dataset-II. The ROC curve depicts the true positive rate (sensitivity) and false positive rate (1-specificity). The larger AUC, the better the classification performance.

#### Class activation map

In addition to performing quantitative evaluations, the decision-making process of the model was also attempted to be understood. For this purpose, the class activation map (CAM) was generated to highlight which region of the image plays a decisive role in the final classification. By CAM, we could observe the contribution of different areas of the image to the final prediction and understand the potential relationship between eye features and diabetes.

#### Comparison with the baseline

Experiments were conducted to verify that the HMT-Net performed better in classifying Q5 with the help of solving tasks Q1 ~ Q4. Figure 2a shows a deep neural network (baseline) that does not utilize (ophthalmologist-designed) Q1 ~ Q4 while using a single $F\text{-}Net$ (ResNet50) to directly predict diabetes (Q5). The diabetes screening performance of this baseline architecture was compared with that of the HMT-Net.

The *F-Net (Q1)* ~ *F-Net (Q4)* were also used individually for detecting diabetes to prove that the combination of these classifiers can improve the detection.

#### Comparison with ophthalmologists

Four ophthalmologists with different qualifications, including two residents, one senior doctor, and one expert, participated in a comparative study. They were different from those who participated in data collection and model development. First, ophthalmologists were trained to learn the typical images of diabetic conjunctival lesions in the training set (Dataset-I), and then determined if the individuals have diabetes based on the conjunctival images in the test dataset (Dataset-II). Each ophthalmologist and model was tested with the same data. The test images were randomly disordered and the relevant information outside the ophthalmologist and the image was hidden to ensure that they could perform independently and impartially. The average test results of the four ophthalmologists were compared with those of the deep learning method.

## Results

### Baseline data of subjects

A total of 200 participants were recruited, and 18 were excluded due to their unqualified medical history or conjunctival lesions. The remaining 182 subjects (97 patients with type 2 diabetes and 85 controls) participated in conjunctival imaging. After dataset cleaning, a total of 611images (405 from 68 diabetic patients and 206 from 62 healthy individuals) were included. We divide the data set into 5 parts (from Set1 to Set5) and then select one part of the sample as the test set each time, and then use the remaining samples for training. The statistics of the divided data set was shown in the Table 1.

**Table 1:** Division of dataset in cross validation.

	Set1		Set2		Set3		Set4		Set5	
	D	H	D	H	D	H	D	H	D	H
number	84	44	71	43	83	44	94	43	73	32

(D = diabetes, H = health).

### Evaluation of Q1 ~ Q4 classification models

In Stage-I, we trained and tested Q1 ~ Q4 using Dataset-I. The performance is summarized in Supplementary Document-p10, Table S1.

### Class activation map (CAM)

**Figure 3:**
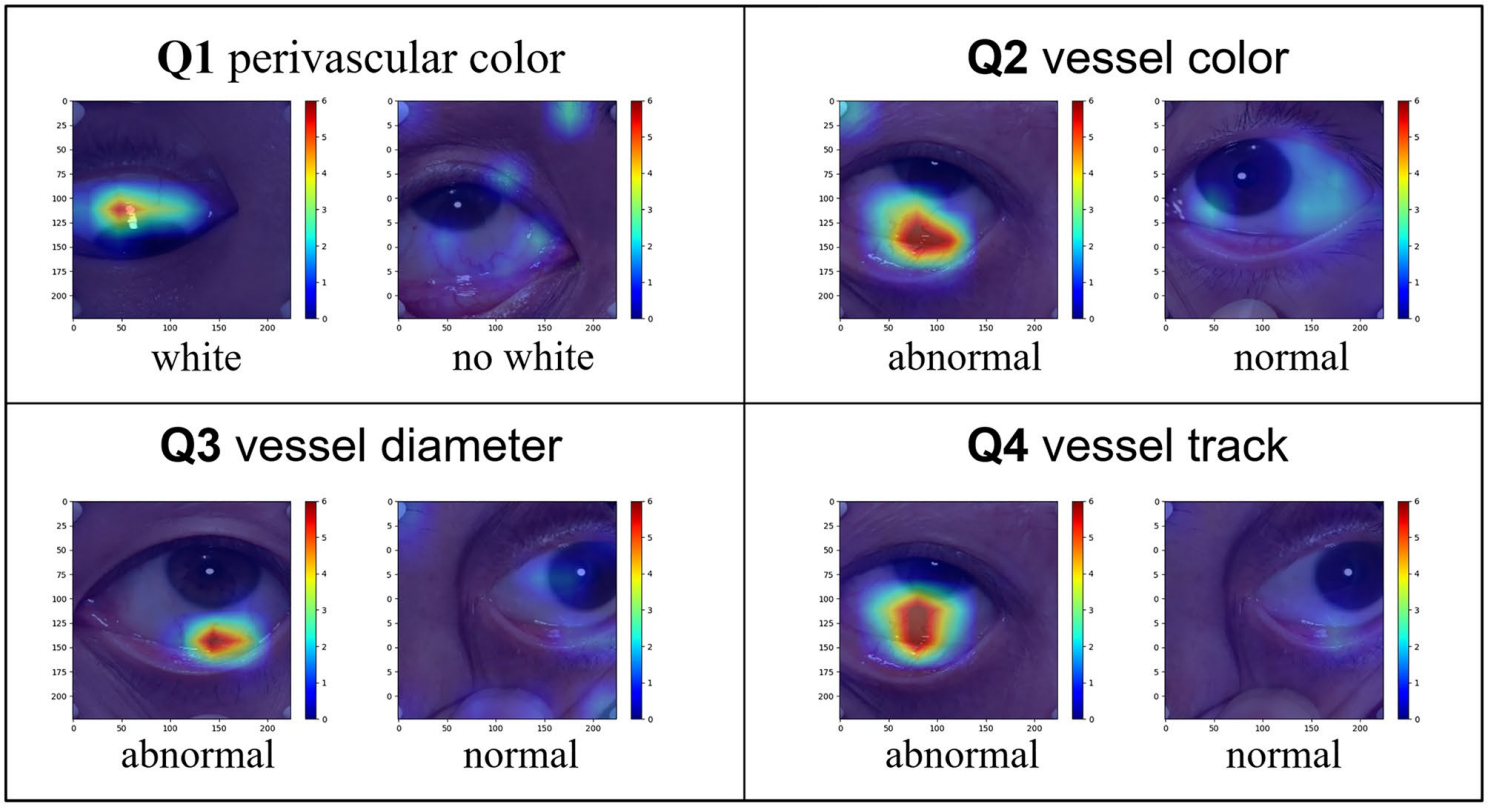
Visualization results of the classification networks of Q1 ~ Q4. Heat maps illustrate which part of the image contributes more to the classification results.

In addition to quantitative performance metrics, we also visualized features extracted by the classification networks of Q1 ~ Q4 on class activation maps. As shown in Fig. 3, brighter colors indicate greater contribution to discrimination. For example, the networks focus more on areas where blood vessels are located. Therefore, in Q2 (vessel color), if the network detects a blood vessel with abnormal color, the region will be highlighted; otherwise, the network will randomly examine other regions with weaker intensity and provide a “normal” prediction. Similar patterns could be observed in the visualization results of Q1, Q3, and Q4.

### Classification results of Q5

**Figure 4:**
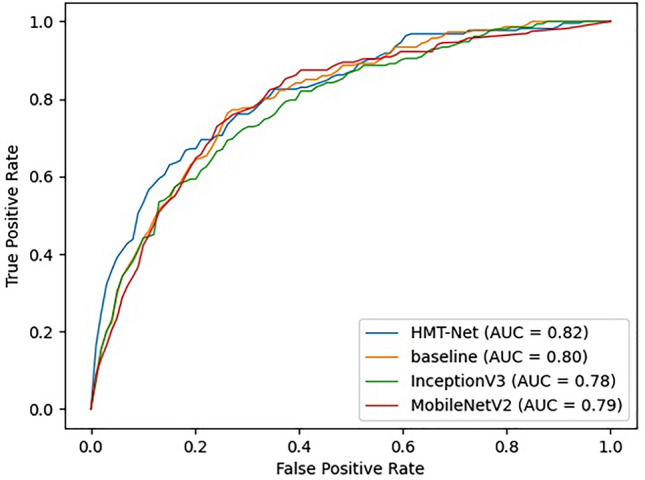
Receiver operating characteristic curves for HMT-Net and other models. AUC = area under the receiver operating characteristic curve.

In Stage-II, the classification algorithms of Q5 were trained and tested on training set and test set of Dataset-II respectively. Sensitivity, specificity, accuracy, and AUC were used to evaluate their performance. For the HMT-Net we designed, a fivefold cross-validation was performed on the newly divided data set, and the results were shown in the Table S2 (Supplementary Document-p11). Each row in the table represents the classification result obtained by using the corresponding part of the data (from Set1 to Set5) as the test set, and the remaining samples as the training set. These metrics comprehensively reflected the screening ability of the HMT-Net. The accuracy, sensitivity, and specificity of the HMT-Net were 79.70%, 69.08%, and 75.15%, respectively. The ROCs for the HMT-Net are shown in Fig. 4, and the average AUC was 0.82.

Table S3 (Supplementary Document-p12) showed the results of using $F\text{-}Net (Q1)$ ~ $F\text{-}Net (Q4)$ individually for detecting diabetes. In this Table (Supplementary S3), the baseline refers to the network that does not use any sub-question to help classify diabetes, and F-Net (Q1) ~ F-Net (Q4) respectively refer to the network that uses each sub-question (Q1 ~ Q4) to help classify diabetes. HMT-Net refers to our comprehensive network that is assisted by all the 4 sub-questions. As shown in this table, compared with baseline, when either one of the four sub-questions is used, the newly learned features are beneficial to the final identification of diabetes: the accuracy and AUC of diabetes classification have been improved. When all the 4 sub-questions are used, the HMT-Net integrates the features from all these sub-questions, and produces the best accuracy and AUC of diabetes classification.

### Comparison with other models

Other methods of diabetes identification were trained and tested following the same way mentioned above. ROCs for different models are shown in Fig. 4: the AUC of the HMT-Net was higher than that of the baseline. A comparison using the quantitative metrics is given in Table 2: the HMT-Net outperformed the baseline in AUC, accuracy, and sensitivity. In comparison with other deep-learning algorithms, the HMT-Net performed better than the baseline, InceptionV3, and MobileNetV2.

**Table 2:** The comparison results between fivefold cross-validation and other algorithms.

	SE (%)	SP (%)	AUC
HMT-Net	78.70	69.08	0.82
ResNet50	74.76	69.57	0.80
MobileNetV2	72.38	73.22	0.79
InceptionV3	78.68	62.80	0.78

SE = Sensitivity, SP = Specificity, ACC = Accuracy, AUC = Area under ROC curve.

### Comparison with ophthalmologists

Compared with the same test set used by ophthalmologists, the accuracy, sensitivity, and specificity of the HMT-Net were 23%, 20%, and 27% higher, respectively (Table 3). The time needed for the HMT-Net to make judgment was shorter than that for the ophthalmologists (1.24 s vs 7.35 s; *P* < 0.001). Moreover, even if the ophthalmologists were trained before the test, they still struggled to identify diabetes based on conjunctival images alone.

**Table 3:** Performance of artificial intelligence vs ophthalmologists in identifying diabetes based on conjunctival images.

	SE (%)	SP (%)	ACC (%)	Speed (s/image)
HMT-Net	78.70	69.08	75.15	1.24
Human average	59.02	42.11	51.59	7.76
ophthalmologist 1	70.10	55.26	63.58	9.21
ophthalmologist 2	62.89	38.16	52.0	6.24
ophthalmologist 3	64.95	18.42	44.51	8.25
ophthalmologist 4	38.14	56.68	46.24	7.35

SE = Sensitivity, SP = Specificity, ACC = Accuracy.

## Discussion

The HMT-Net model designed in the present study can locate several associations between conjunctival images and diabetes, which provides the possibility for automatic screening of diabetes by deep learning analysis of conjunctival images in the future. According to literature review, this is the first study using a deep learning algorithm to analyze conjunctival images for diabetes recognition.

This study has several advantages. First, the HMT-Net model only uses conjunctival images for diabetes recognition, which may provide a new method for diabetes screening in the absence of special medical equipment in the future. Second, the model can automatically process data without manual calculation, which is more efficient. Moreover, the algorithm is more accurate than ophthalmologists in terms of diagnostic accuracy (Table 3). Third, developers creatively use the problem-oriented training network architecture to effectively overcome the problem of insufficient datasets, which can be extensively applied to other fields.

In addition, the model has two significant medical applications. First, the HMT-Net model can be used to detect microangiopathy in early diabetes mellitus. Early detection is crucial for decreasing the risk of disease progression and improving prognosis. Previous studies have found that conjunctival abnormalities occur earlier than retinal abnormalities in patients with diabetes^14,23^. This has been further confirmed by the current study. More than half of the diabetic patients included in this study were at the early stages of the disease who did not develop diabetic retinopathy, which can be identified by the HMT-Net model. This finding suggests that the HMT-Net model may be used for early screening of diabetes. Second, the HMT-Net model has great potential in rapid, non-invasive, and remote screening of diabetes. Approximately 4.2 million deaths were caused by diabetes and its complications in 2019 globally^5^. Regular detection and timely referrals are crucial for reducing the burden of diabetes. Although regular blood tests can detect blood sugar increase in time, the lack of awareness of physical examination, traumatic blood tests, and uneven distribution of medical techniques have led to approximately 37.8%-59.7% of diabetic patients in the world without early diagnosis^5^. A simple, non-invasive, remote diagnostic model needs to be implemented to solve this problem, which is shown by the results of this study.

It is recognized that hyperglycemia can cause pathological changes of blood vessels throughout the body, and its pathogenesis can be summarized as follows: Hyperglycemia causes oxidative stress and rheological changes in blood vessels, which may lead to adverse changes in the flow properties of red blood cells by changing the composition and physical properties of erythrocyte membranes, such as deformability, aggregation, and adhesion to endothelium, significantly promoting blood flow silt. The changes of rheological factors, especially due to the change of erythrocyte deformability, contribute to shear stress alteration and the pathogenesis of hyperglycemic vascular disease. A large number of previous studies have observed such vascular changes through retina. However, both fundus photography and OCTA rely on expensive and large fundus imaging equipment. Conjunctival vessels can be observed by naked eyes, therefore, conjunctival images require no special equipment but need to be clearly photographed and magnified for analysis. Conjunctival vessels are 5–70 μm in diameter and belong to microvessels, which can reflect the vascular complications of diabetes^24^. Cheung et al.^7^ used CAIM and found that microvascular abnormality severity index was associated with HbA1c levels in T1DM. Based on previous studies^13,15,23,25,26^, impairment of vascular barrier function and deposition of hemosiderin resulted in abnormal perivascular color. Changes in the conjunctival vessels of diabetes patients include thickening of vascular wall, abnormal perivascular color, morphological distortion, microaneurysms, abnormal A/V ratio, slower blood flow, etc., which are generally difficult to be detected by eyes. In 1954, Drtzel et al. first identified specific changes of the conjunctiva in diabetic patients^23^. Later in 1956, Drtzel et al. further described the conjunctival changes on angiography images and their relationship with aging in Circulation magazine^13^. In 2001, Cheung et al. identified the presence of microvascular abnormalities in the conjunctival microcirculation of patients with T2DM by computer-assisted intravital microscopy (CAIM)^25^. In 2009, Cheung et al. found that real-time in vivo microvascular abnormalities should correlate with biochemical markers of inflammation/endothelial dysfunction in T1DM^7^. In 2011, Cheung et al. provide evidence that significant vasculopathy had developed in the microcirculation in the conjunctiva, although diabetic retinopathy had not developed significantly in the same patients^6^. In 2015, DeBuc et al. firstly presented the feasibility and applicability in diagnostic imaging of conjunctival microangiopathy in a diabetic patient using a commercially available Retinal Function Imager (RFI)^10^. In 2016, Shahidi et al. used ordinary least square regression and Fisher linear discriminant analysis assess conjunctival microvasculature image^9^. In 2017, Khansar et al. published a study on the different conjunctiva changes that occur in diabetic patients in Scientific Reports^27^. In 2017, Khan also suggested et al. “a clinical correlation of conjunctival microangiopathy with grades of retinopathy in type 2 diabetes mellitus”^14^. The analytical methods used in these studies, however, are time-consuming and insufficiently objective; and this limits their potential to be developed into diagnostic indicators. In this work, we aim to solve this problem via deep learning. Currently, the identification rate by the doctors using conjunctival images is only around^9^. This is because the correlation between conjunctival images and diabetes need to be discovered through complex measurement, calculations, and comparisons, and is difficult to identify directly with the naked eyes. Artificial intelligence through multiple learning and training can help better identify nuances that are not easily perceived by human beings, and more effectively aggregate them for the targeting classification task. In this work, with our deep learning pipeline, this accuracy rate has now increased to 75.15%. Furthermore, with the growth of the collected data set, we believe the accuracy of such a system can be further improved. In the future, the HMT-Net model can be used as an opportunistic screening tool, integrated into general ophthalmology, and even applied in mobile phone software, which is convenient to obtain general health suggestions.

In this work, we extracted four groups of features (Q1 ~ Q4) from easier sub-problems. From the classification results of Q1 ~ Q4, our method performs well in the classification of Q1 ~ Q4 (sensitivity, specificity, and accuracy are all above 70%, Supplementary Document-p10, Table S1). In addition, CAM visualization shows that the networks focus on suitable regions when performing the inference. The model can classify the regions of interest and potential features according to the ophthalmologist's experience, which also means that the ophthalmologist can interpret the decision made by the model by examining the suggested area of interest. In turn, the model can also examine the reliability of the classification results; using the relevant features of Q1 ~ Q4, the model provides the meaningful classification of conjunctival regions, rather than random classification. Therefore, the corresponding feature extractors ($F\text{-}Net (Q1)$ ~ $F\text{-}Net (Q4)$) can be obtained from the Q1 ~ Q4 classification model, which serves as a crucial prerequisite for building a network using limited images.

The learning strategy used in this study is different from commonly adopted direct end-to-end pipeline in many medical classifications. Specifically, we combined the professional knowledge of ophthalmologists and realized the classification of a difficult problem through a hierarchical multi-task network, which is built upon sub-networks that are trained to answer simpler classification questions Q1 ~ Q4. In the fivefold cross-validation (Supplementary Document-p11, Table S2), each time a different cross-validation is performed, the results of the classification will fluctuate to a certain extent. The performance of the Set3 classification network is poor. The reason may be that the mean arterial pressure of Set3 patients is lower than that of other groups (as shown in the Supplementary Table S4). Blood pressure is also an important factor affecting vascular morphology. The higher the blood pressure, the easier it is to develop vascular lesions, which is similar to that caused by diabetes. Thus, the detection of set3 is relatively difficult. In general, from the average results of fivefold cross-validation, the average AUC of the classification of the HMT-Net we designed can reach 0.82, which demonstrates a good classification performance.

In this study, we designed a novel effective data augmentation strategy based on the characteristics of conjunctival images. The valid region of blood vessels is usually located in the central area of the image, which is more critical in feature learning. Therefore, we located these regions through segmentation, and then added noise in other areas to improve the training robustness. Meanwhile, similar to Hwang’s work^28^, we also expanded the size of the training sets via general augmentation methods such as rotation and flipping. Through these data augmentation strategies, the network achieved an improved generalization ability.

## Limitations of the study

The study has some limitations. First of all, though the current study is of certain significance, it has a small sample size with single center and single race. But at the same time, it also reflects the advantages of the HMT-Net model: This algorithm can overcome data insufficiency. In clinical research, especially in such early exploratory research, it is difficult to collect thousands of images at once, resulting in the network can not learn effectively, which undoubtedly limits the combination of artificial intelligence and medicine. If the method of this study is recognized, it will have great application value. Furthermore, the HMT-Net model has an accuracy of 75.15%. Besides the lack of training, we think, this discrepancy is also related to the ocular surface. In a multicenter study involving thousands of ocular surface images in identifying hepatobiliary diseases^3^, the accuracy of identification was only 74% (71%–76%), which is almost similar to our results. Although the HMT-Net model has low specificity, its sensitivity to diabetes detection is high. We believe that the effectiveness and versatility of the model can be continuously improved with upgrading artificial intelligence and dataset collections.

## Conclusion

In this study, we proposed a novel HMT-Net artificial intelligence model, based on expert knowledge fusion and a deep classification network, to detect diabetes on conjunctival images. Our findings indicate a high precision accuracy for the detection of diabetes in relation to an expert ground truth. The advantage of this system is that it does not require a large dataset to train the model. It is also transparent, easy to interpret, and can be used to facilitate the visualization of intermediate processes. This exploratory study provides the basis for the development of a remote mobile screening tool for diabetes in the future. In the near future, we will collect and prepare a larger dataset to further validate and improve the performance of our deep learning model. Another future direction of our work is to reduce the memory and computational cost of the pipeline so that the current model can be deployed on mobile platforms.

## Supplementary Information

Supplementary Information.

## Data Availability

Data underlying the results presented in this paper are not publicly available at this time but may be obtained from the authors upon reasonable request.

## References

[1] Mitani A (2020). Detection of anaemia from retinal fundus images via deep learning. Nat. Biomed. Eng..

[2] Poplin R (2018). Prediction of cardiovascular risk factors from retinal fundus photographs via deep learning. Nat. Biomed. Eng..

[3] Xiao W (2021). Screening and identifying hepatobiliary diseases through deep learning using ocular images: a prospective, multicentre study. Lancet Digit. Heal..

[4] Banbury C (2020). Spectroscopic detection of traumatic brain injury severity and biochemistry from the retina. Biomed. Opt. Express..

[5] International Diabetes Federation. *IDF diabetes atlas*. 9th edn, (International Diabetes Federation, 2019).

[6] To WJ, Telander DG, Lloyd ME, Chen PC, Cheung AT (2011). Correlation of conjunctival microangiopathy with retinopathy in type-2 diabetes mellitus (T2DM) patients. Clin. Hemorheol. Microcirc..

[7] Cheung AT (2009). Correlation of microvascular abnormalities and endothelial dysfunction in Type-1 Diabetes Mellitus (T1DM): a real-time intravital microscopy study. Clin. Hemorheol. Microcirc..

[8] Khansari MM, Wanek J, Felder AE, Camardo N, Shahidi M (2016). Automated assessment of hemodynamics in the conjunctival microvasculature network. IEEE Trans. Med. Imaging.

[9] Khansari MM (2016). Automated fine structure image analysis method for discrimination of diabetic retinopathy stage using conjunctival microvasculature images. Biomed. Opt. Express..

[10] Stuebiger N, Smiddy W, Wang J, Jiang H, DeBuc DC (2015). Assesment of conjunctival microangiopathy in a patient with diabetes mellitus using the retinal function imager. J. Clin. Exp. Ophthalmol..

[11] Panayides AS (2020). AI in medical imaging informatics: current challenges and future directions. IEEE J. Biomed. Health Inform..

[12] Shin H-C (2016). Deep convolutional neural networks for computer-aided detection: CNN architectures, dataset characteristics and transfer learning. IEEE Trans. Med. Imaging..

[13] Ditzel J (1956). Angioscopic changes in the smaller blood vessels in diabetes mellitus and their relationship to aging. Circulation.

[14] Khan MA (2017). A clinical correlation of conjunctival microangiopathy with grades of retinopathy in type 2 diabetes mellitus. Med. J. Armed Forces India.

[15] Khansari MM, Tan M, Karamian P, Shahidi M (2018). Inter-visit variability of conjunctival microvascular hemodynamic measurements in healthy and diabetic retinopathy subjects. Microvasc. Res..

[16] Khansari MM (2016). Automated fine structure image analysis method for discrimination of diabetic retinopathy stage using conjunctival microvasculature images. Biomed. Opt. Express.

[17] He, K., Zhang, X., Ren, S. & Sun, J. In *Proceedings of the IEEE conference on computer vision and pattern recognition.* 770–778.

[18] Simonyan, K. & Zisserman, A. In *IEEE Conference on Computer Vision & Pattern Recognition.* (IEEE).

[19] Howard, A. G. *et al.* In *IEEE Conference on Computer Vision & Pattern Recognition.* (IEEE).

[20] Deng, J., Dong, W., Socher, R., Li, L. J. & Li, F. F. In *IEEE Conference on Computer Vision & Pattern Recognition.*

[21] Hu, J., Shen, L., Albanie, S., Sun, G. & Wu, E. In *IEEE Transactions on Pattern Analysis and Machine Intelligence.* 7132–7141 (IEEE). 10.1109/TPAMI.2019.2913372 31034408

[22] Paszke, A. *et al.* In *Advances in neural information processing systems.* 8026–8037.

[23] Ditzel J, Sagild U (1954). Morphologic and hemodynamic change in the smaller blood vessels in dibetes melllitus. N Engl. J. Med..

[24] Wang L (2016). Vessel sampling and blood flow velocity distribution with vessel diameter for characterizing the human bulbar conjunctival microvasculature. Eye Contact Lens Sci. Clin. Pract..

[25] Cheung AT, Ramanujam S, Greer DA, Kumagai LF, Aoki TT (2001). Microvascular abnormalities in the bulbar conjunctiva of patients with type 2 diabetes mellitus. Endocr. Pract..

[26] Khan MA (2017). A clinical correlation of conjunctival microangiopathy with grades of retinopathy in type 2 diabetes mellitus. Med. J. Armed. Forces India.

[27] Khansari MM (2017). Assessment of conjunctival microvascular hemodynamics in stages of diabetic microvasculopathy. Sci. Rep..

[28] Hwang DK (2019). Artificial intelligence-based decision-making for age-related macular degeneration. Theranostics.

