# Supplementary material for: Identifying diabetes from conjunctival images using a novel hierarchical multi-task network
Source: Sci Rep. 2022 Jan 7;12:264. doi: 10.1038/s41598-021-04006-z (PMC8742044; doi:10.1038/s41598-021-04006-z)
Supplement: Supplementary file 1 — Supplementary Information. [file 41598_2021_4006_MOESM1_ESM.docx]

**Supplemental Document**

**Exclusion criteria**

(1) patients with a history of diseases affecting conjunctival vessels (such as hypertension and sickle cell anemia);

(2) patients who wore contact lenses and underwent ophthalmic surgery within 3 months;

(3) patients with conjunctival abnormalities caused by eye diseases.

**Image acquisition**

The eyes of all the selected subjects were examined in detail. All the images were taken by the same technician using a digital camera (EOS 5D Mark II, Canon, Tokyo, Japan) in the same darkroom environment. When taking conjunctival photos, a head frame similar to a slit lamp (for fixing only) and a 15-cm board were used to ensure a constant shooting distance from the lens to the conjunctiva. Fixed shooting brightness, light source position (camera flash), and magnification of 10 times were used. Before filming, the subjects were asked to close their eyes and sit still for at least five minutes. The subjects then put their head on the headframe, opened the right eyelid with two fingers, fully exposed the bulbar conjunctiva, and then rotated the eyeball upward, downward, left, and right according to the researchers' instructions. The researchers took pictures in every direction to ensure that the focus was on the bulbar conjunctiva and then pressed the shutter. The left eye was taken in the same way.

**Image labeling process**

The image labeling process was performed as follows. We first designed four related sub-problems (Q1~Q4) based on previous studies on the characteristics of conjunctival lesions in diabetes. We then selected various modern computer measuring tools, including the RGB digital colorimeter tool for marker classification based on color value and a digital ruler to determine the blood vessel diameter. These measured parameters were subsequently combined with different classification criteria, as shown in Figure S1 in Supplementary document. Three ophthalmologists were subsequently trained to assess the ophthalmic images based on these criteria. The ophthalmologists had to achieve a consistency level of 99% on the same label of the same picture. If this level of consistency was not achieved, the classification criteria were again discussed, and the doctors were re-assessed until the required standard was achieved. After reaching the required standard, the three doctors tagged all the images independently. If the labeled results given by the three doctors were consistent, the object marking was tagged as successful. In cases of disagreement, the doctors discussed the images until a consensus was reached and the object marking was tagged as successful. If the doctors failed to reach an agreement, the image was marked as controversial and removed from the training dataset.


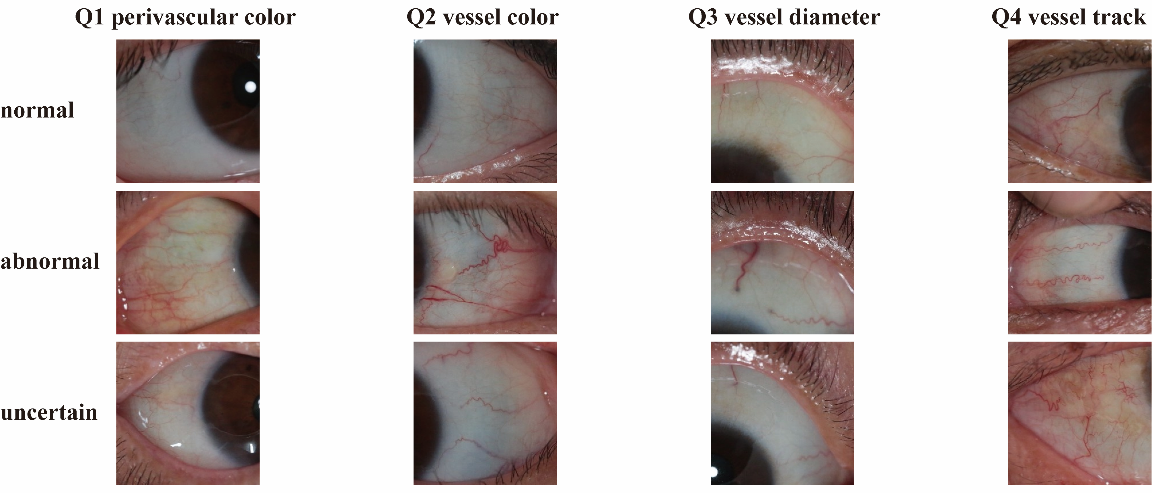


**Figure S1. The classification criteria of Q1 ~ Q4.** Q1 (perivascular color) Normal: Meet the following two criteria: (1) the value of the color RGB is close to red: green: blue = 1:1:1; (2) the sum of all these areas ≥ 2/3 total study area. Abnormal: More than 2/3 areas are obvious no-white. Uncertain: Does not meet the previous criteria. The digital colorimeter on the computer was used to measure the RGB value of the pixel. Q2 (vessel color) Normal: The value of the color RGB of the most obvious vessel is close to red: green: blue = 2: 0.8: 0.8. Abnormal: The value of the color RGB of the most obvious vessel is close to red: green: blue = 2: 1: 1. Uncertain: Does not meet the previous criteria. The digital colorimeter on the computer was used to measure the RGB value. Q3 (vessel diameter) Normal: The most obvious vessel is thinner than the root of the eyelash. Abnormal: At least one of the following criteria is fulfilled: (1) one vessel has a diameter close to the root of the eyelash and a length exceeding 1/2 of the distance from its root to the limbus; (2) more than 2/3 vessels have a diameter close to the root of the eyelash. Uncertain: Does not meet the previous criteria. Q4 (vessel track) Normal: Vessels are relatively straight. Abnormal: At least one of the following criteria is fulfilled: (1) one vessel has tortuosity like a continuous “m” or “s” and a length exceeding 1/2 of the distance from its root to the limbus; (2) more than 2/3 vessels have tortuosity. Uncertain: Does not meet the previous criteria.

**Image preprocessing**

Based on the characteristics of the captured conjunctival images, we found that the effective area of the blood vessels was typically located in the central area of the image. Accordingly, the original input images were cropped to remove boundary regions. More specifically, a 960 * 640 pixel^2^ original image was cropped to 500 * 300 pixel^2^. As shown in Figure S2, for the cropped image, through image graying, morphological operation, threshold segmentation, and other image processing algorithms, the mask of the eye white area in the conjunctival image was obtained. During this process, the image that cannot be successfully masked was excluded.

**
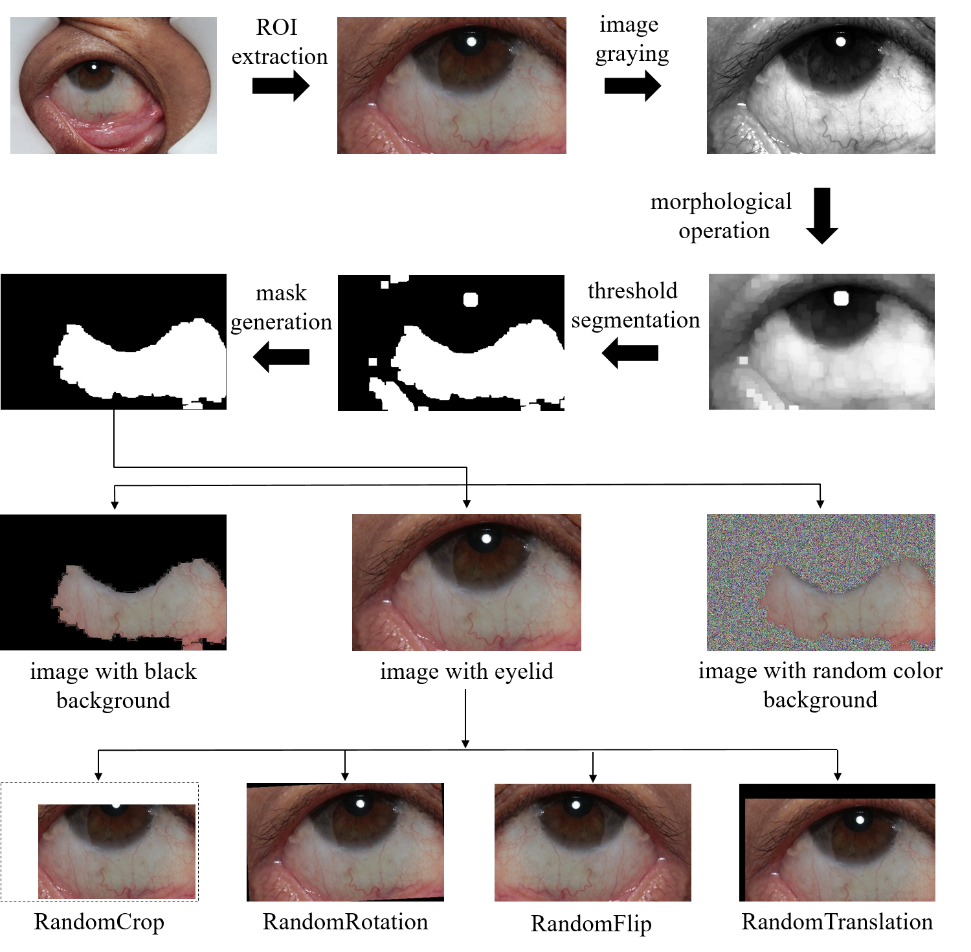
**

**Figure S2. Process of image preprocessing and data augmentation. ROI = region of interest.**

**Data augmentation**

For each image in the training set, an image with a black background and an image with random color background were generated based on the original image. As shown in Figure S2, based on the generated mask, the noise was added outside the valid area to generate simulated training images. Two methods were employed to add noise: 1) adding simple noise - the RGB values of pixels outside the valid area were (255, 255, 255); 2) adding complex noise - the RGB values of pixels outside the valid area were random within (0, 255). The labels of the generated images were the same as those of the original images. The original and generated images were mixed to form the training set. By adding more complex noises outside the valid area in the input image, the network was tuned to focus more on the valid area for classification. The network with stronger generalization ability, which was better adapted to the application scenarios, was obtained by transforming data augmentation of the training images.

Besides, to further improve the generalization ability of the network, several general data augmentation techniques were carried out, including 1) randomly cropping the image with 80% of the original image size; 2) randomly rotating the image within a range of ±5°; 3) horizontally and vertically flipping the image with 50% probability; 4) randomly translating the image within ±20% of the original image size1.

- ROI extraction: ROIs were extracted according to the position of eyes in the image. A 960 * 640 pixel^2^ original image was cropped to 500 * 300 pixel^2^.
- Image graying: ROI processing methods included grayscale transformation, histogram equalization, and median filtering (the size of filter was 1).
- Morphological operation: four consecutive eroding and dilating were performed (the size of structure element was 5 * 5).
- Threshold segmentation: the image was segmented with a threshold of 180. The gray value of pixels greater than 180 was set to 255 and that of pixels less than or equal to 180 was set to 0.
- Mask generation: all contours were detected with an area of 100 to 1000, and the largest contour was used as the contour of conjunctiva. The value of pixels inside the contour was set to 255 and that of pixels outside the contour was set to 0 for obtaining the mask.
- Add noise: added noise outside the valid area based on mask. 1) simple noise, set the RGB values of pixels outside the valid area to (255, 255, 255); 2) complex noise, set the RGB values of pixels outside the valid area to random values within [0, 255].
- General data augmentation:

1. cropped the image randomly with 80% of the original image size;
2. rotated the image randomly within a range of ±5°;
3. horizontally and vertically flipped the image with 50% probability;
4. translated the image randomly within ±20% of the original image size.

We have added a comparative experiment where data enhancement is not used, and the results are shown in the following table.

|  | SE (%) | SP (%) | AUC |
| --- | --- | --- | --- |
| No augmentation | 65.71 | 74.98 | 0.79 |
| With augmentation | 78.70 | 69.08 | 0.82 |

Based on this experiment, we can see that with augmentation, the sensitivity of the classification improves from 66% to 79%, while the specificity of classification decreases from 75% to 69%. The overall AUC improves from 79% to 82%.

**Supplementary Methods**

The model was trained and tested on a server with an operating system of Ubuntu 18.04 and a GPU of RTX 2080 Ti.

Backbone: ResNet50

Adam optimization algorithm with $\beta_{1}$=0.9, $\beta_{2}$=0.999, =e-8 and a batch size of 64 was used.


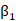

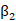

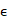


The initial learning rates of the classification networks of Q1 ~ Q4 and the HMT-Net were set to 5e-4 and 5e-6 respectively, and 150 epochs were sufficient to converge.

The trend of loss during training is shown in the Figure S3

**
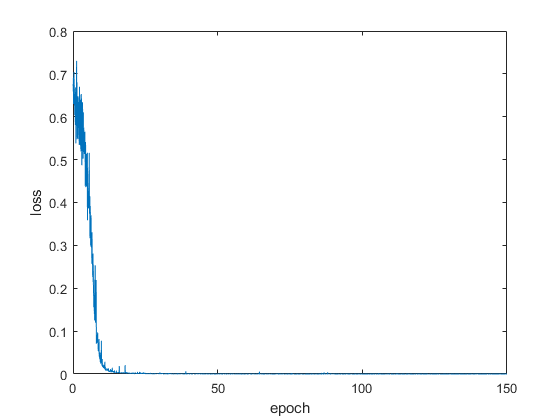
Figure S3. Illustration of the changes of the loss function value.**

The use of ResNet-50 as a backbone resulted in a very large model (733.8 M). Unfortunately, this model is too large to be used on mobile phones. Therefore, for future work, we are planning to evaluate model compression technologies to reduce the memory and computational cost of the algorithm.

Supplementary Table S1. Classification results of Q1 ~ Q4

|  | SE (%) | SP (%) | ACC (%) |
| --- | --- | --- | --- |
| Q1 | 83.57 | 70.20 | 78.65 |
| Q2 | 87.23 | 89.20 | 88.25 |
| Q3 | 81.38 | 84.29 | 82.68 |
| Q4 | 85.14 | 75.79 | 79.35 |

SE=Sensitivity, SP= Specificity, ACC= Accuracy.

Supplementary Table S2. HMT-Net performance in identifying diabetes using 5-fold cross-validation.

|  | SE (%) | SP (%) | AUC |
| --- | --- | --- | --- |
| Set1 | 85 | 73 | 0.84 |
| Set2 | 90 | 53 | 0.83 |
| Set3 | 63 | 68 | 0.70 |
| Set4 | 90 | 70 | 0.89 |
| Set5 | 66 | 81 | 0.84 |
| average | 79 | 69 | 0.82 |

SE=Sensitivity, SP= Specificity, ACC= Accuracy, AUC= Area under ROC curve.

Supplementary Table S3. The performance of using F-Net (Q1) ~ F-Net (Q4) individually for detecting diabetes

|  | SE (%) | SP (%) | ACC (%) | AUC |
| --- | --- | --- | --- | --- |
| Baseline | 74.76 | 69.57 | 72.90 | 0.80 |
| F-Net(Q1) | 77.11 | 67.22 | 72.84 | 0.81 |
| F-Net(Q2) | 78.16 | 67.57 | 74.14 | 0.81 |
| F-Net(Q3) | 74.32 | 73.46 | 73.52 | 0.81 |
| F-Net(Q4) | 79.73 | 65.62 | 74.27 | 0.80 |
| HMT-Net | 78.70 | 69.08 | 75.15 | 0.82 |

SE=Sensitivity, SP= Specificity, ACC= Accuracy, AUC= Area under ROC curve.

| Mean arterial pressure | Average value  (mmHg) | Median  (mmHg) | P-Value  vs Set3 |
| --- | --- | --- | --- |
| Set1 | 94.81 | **98.33** | 0.35 |
| Set2 | 108.30 | **103.67** | 0.99 |
| Set3 | 101.36 | **97.33** | - |
| Set4 | 106.04 | **109.33** | 0.01 |
| Set5 | 110.05 | **107** | 0.00 |

**Supplementary Table S4. The mean arterial pressure of each set.**
